# Rapid Implementation of Telerehabilitation for Pediatric Patients During Covid-19

**DOI:** 10.5195/ijt.2021.6371

**Published:** 2021-06-22

**Authors:** Rachel Bican, Catie Christensen, Kristin Fallieras, Grace Sagester, Sara O'Rourke, Michelle Byars, Kelly Tanner

**Affiliations:** 1 Division of Clinical Therapies, Nationwide Children's Hospital, Columbus, Ohio, USA; 2 School of Health and Rehabilitation Sciences, College of Medicine, the Ohio State University, Columbus, Ohio, USA

**Keywords:** Covid-19, Occupational Therapy, Pediatrics, Physical Therapy, Telehealth, Telerehabilitation

## Abstract

The COVID-19 pandemic necessitated a sudden limitation of in-person outpatient occupational and physical therapy services for most patients at a large, multisite pediatric hospital located in the Midwest, United States. To ensure patient and staff safety, the hospital rapidly shifted to deliver most of these services via telerehabilitation. The purposes of this study were to (1) describe the rapid implementation of telerehabilitation during the COVID-19 pandemic, (2) describe the demographic characteristics of patients who continued in-person services and those who received telerehabilitation, and (3) evaluate the therapists' perceptions of telerehabilitation for physical and occupational therapy. Most of the children (83.4% of *n*=1352) received telerehabilitation services. A family was more likely to choose to continue in-person visits if their child was <1-year-old, had a diagnosis of torticollis, received serial casting, or was post-surgical. Occupational and physical therapy therapists (*n*=9) completed surveys to discern their perceptions of the acceptability of telerehabilitation, with most reporting that telerehabilitation was as effective as in-person care.

Telerehabilitation has been proposed as a potentially effective model of care for pediatric populations (Camden et al., 2020; Olson et al., 2018; Shigekawa et al., 2018; Tanner et al., 2020; Tenforde et al., 2017, 2020). However, barriers to implementation, including payor reimbursement, perceived and actual technology barriers, liability concerns, and privacy concerns, have prevented routine adoption of this service delivery model (Brophy, 2017; Dorsey & Topol, 2016; Lee et al., 2018; Olson et al., 2018; Sauers-Ford et al., 2019; Tomines, 2019). The Covid-19 pandemic, a public health crisis, accelerated the use of telerehabilitation for pediatric populations to adhere to social distancing guidelines, prevent disruption to an established plan of care, and ensure access to essential therapeutic services (Badawy & Radovic, 2020; Ben-Pazi et al., 2020; Burke et al., 2015; Tomines, 2019).

Previous literature has shown telerehabilitation can be utilized to provide outpatient services for pediatric and adult patients and can be an effective, efficient, and affordable model of care (Burke et al., 2015; Cramer et al., 2019; Levy et al., 2015; Olson et al., 2018; Tenforde et al., 2020). The American Occupational Therapy Association and American Physical Therapy Association both support the use of telerehabilitation (i.e., telehealth) for clinical care, stating that telerehabilitation can be effective in improving patient outcomes and can be flexibly delivered to meet the needs of the patients and their families (American Occupational Therapy Association, 2018; American Physical Therapy Association, 2019).

The effectiveness and applicability of telerehabilitation may vary by pediatric specialty, setting, and patient preference (Tomines, 2019). Studies have shown that telerehabilitation may be effective for pediatric rehabilitation, primary care, and mental health treatment (Gloff et al., 2015; Nelson & Sharp, 2016; Tomines, 2019). Telerehabilitation has also been shown to be feasible and effective in treating children with cerebral palsy (Reifenberg et al., 2017) and autism spectrum disorder (Little et al., 2018). However, since telerehabilitation has not been routinely adopted for pediatric occupational and physical therapies, there is currently no consensus on which patient populations may best be served by telerehabilitation throughout or beyond the COVID-19 pandemic.

The COVID-19 pandemic led to a sudden limitation of in-person outpatient therapy services for most patients at Nationwide Children's Hospital (Tanner et al., 2020). To ensure patient and staff safety, the hospital had to rapidly and unexpectedly prepare to deliver these services via telerehabilitation (Tanner et al., 2020). Therefore, the purposes of this study were to (1) describe the rapid implementation of telerehabilitation during the COVID-19 pandemic, (2) describe the demographic characteristics of patients who continued in-person services and those who received telerehabilitation, and (3) evaluate the therapists' perceptions of telerehabilitation for physical and occupational therapy at a large, multisite pediatric hospital located in the Midwest, United States.

## METHODS

## PARTICIPANTS

The procedures for this retrospective, observational study were approved by the Institutional Review Board of Nationwide Children's Hospital (STUDY00001188). All patients receiving developmental occupational or physical therapy at Nationwide Children's Hospital, a large, urban, multisite pediatric hospital system, between April 1, 2020 and April 30, 2020 were included in this study. Of note, a stay-at-home order was issued for the state of Ohio on March 18, 2020 in response to the COVID-19 pandemic. During this time, both in-person clinic visits and telerehabilitation were available. Patients were recommended for in-person clinic visits if they met one or more of the following criteria: (1) less than one year old, (2) had torticollis, (3) were currently receiving, or were planning to receive, serial casting, (4) had recently been discharged from inpatient rehabilitation or had surgery, or (5) had no access to technology required for telerehabilitation. These criteria were developed by the Developmental Physical and Occupational Therapy Program Managers with input via email from all staff members. For patients that met the criteria for in-person clinic visits, families ultimately were able to decide whether to continue in-person visits or transition to a telerehabilitation service delivery model. If a child did not meet the criteria for in-person visits, they were offered services through telerehabilitation or placed on-hold based on family preference.

## PROCEDURES

### IMPLEMENTATION OF TELEHEALTH

Over a three-week period, the hospital developed and implemented a strategic plan for the rapid uptake of services provided through telerehabilitation, including preparation for telerehabilitation and telerehabilitation roll-out to ensure a successful transition from an in-person to a telerehabilitation service delivery model. This rapid implementation was carried out by a multidisciplinary team that included the director of rehabilitation services, program managers, clinical leaders, performance improvement staff, evidence-based practice and research coordinators, information technology staff, scheduling support staff, physical and occupational therapists, and therapy aides.

### PREPARATION FOR TELEREHABILITATION

The first component for telerehabilitation preparation was to obtain, organize, and train all staff on technology and documentation. The hospital developed two methods of telerehabilitation service delivery: billable telephone calls and video sessions via a secure Zoom video platform (Zoom, 2020). Telephone calls were completed through the therapist's work phone. Video telerehabilitation occurred through Zoom, a web-based video conferencing program, hosted by Epic Systems (Software | Epic, n.d.), the electronic medical records system utilized by the hospital. The sessions were Health Insurance Portability and Accountability Act (HIPAA) compliant, encrypted, and password protected. All telerehabilitation documentation included caregiver consent and followed payor guidelines.

Next, the hospital developed specific messaging to caregivers and families regarding telerehabilitation. The treating therapist initiated the first contact with the family via phone with the goals to provide information on the upcoming switch to telerehabilitation, determine if the family was willing to participate, and decide the most appropriate method of telerehabilitation service delivery according to the family's needs. A written script was developed to guide therapists in this discussion with families. Parents were provided with background information on telerehabilitation, supporting evidence for this service delivery model, and details on how it may be customized for each child.

Finally, all staff members were educated on telerehabilitation best practices based on the available literature, using both formal and informal methods. Formal methods included mandatory in-service training, journal clubs, and shared resources. Informally, staff members were encouraged to ask questions and provide feedback via email and in-person. This staff education emphasized normalizing services provided through telerehabilitation, highlighting research on telerehabilitation, providing a step-by-step guide for conducting a telerehabilitation therapy session, identifying the location of additional resources, and having adequate time for discussion and case examples. Staff education was provided in the two weeks prior to initiating services through telerehabilitation, as well as on an ongoing basis during the delivery of these services.

### TELEREHABILITATION ROLL-OUT

The leadership team at the hospital prioritized the rapid uptake of services through telerehabilitation to promote the safety and well-being of patients, families, and staff during a public health crisis, as well as to minimize any lapses in meaningful and therapeutically necessary patient care.

These goals allowed the department to support therapists to refocus their treatment sessions using the World Health Organization's International Classification of Functioning, Disability, and Health to prioritize the domains of Activities and Participation, rather than Body Functions and Structures (*WHO | International Classification of Functioning, Disability and Health (ICF)*, n.d.). This framework emphasized the prioritization of functional, participation-based goals to allow therapists to provide meaningful services in the child's natural environment.

When we first began using telerehabilitation, we shifted from providing direct patient care to using a hybrid consultation/coaching model via telerehabilitation. We emphasized a parent coaching model to support caregivers navigating new challenges to their family's disrupted daily routine, allowing flexibility for families that may not have access to technology for video visits or with a child who had difficulty attending to a therapist on a screen. Parent coaching also provided an evidence-based framework to support therapists with their transition to telerehabilitation sessions (Little et al., 2018; Baldwin et al., 2013; Novak et al., 2020). The framework for parent coaching included capitalizing on the family's authentic context by integrating the family's interests and routines in treatment sessions, fostering a relationship between the caregiver and child, building in strategies for reflection and feedback, and collaborating with the caregiver to establish joint plans (Little et al., 2018; Wallisch et al., 2019).

## MEASURES

### PATIENT DEMOGRAPHIC INFORMATION

Demographic information was extracted from Epic using the Information Data Enterprise Application (IDEA). IDEA gives users the ability to build queries on the appointment, visit, account, and demographic data from within the electronic medical record for analysis and reporting. Report dates were April 1, 2020 – April 30, 2020. Data extracted included the following: (1) date of visit, (2) patient age at visit (years), (3) rehabilitation type (physical or occupational therapy), (4) service delivery type (clinic or telerehabilitation), (5) diagnosis (ICD-10), (6) patient language, and (7) patient zip code and city of residence. Patient ICD-10 *referral* diagnoses were then categorized into 13 groups for analysis (Table 1). If a child had multiple diagnoses, the primary diagnosis was used for classification. Zip codes were classified using the 2013 National Center for Health Statistics Urban-Rural Classification Scheme for Counties (DD Ingram & SJ Franco, n.d.). This classification includes six levels based on location to a city and population size: large central metro, large fringe metro, medium metro, small metro, micropolitan, and noncore.

**Table 1:** Diagnosis Group

Patients (N)	Diagnosis Group	Description of Diagnosis Group
207	Abnormalities in physiological development	Any diagnosis that involved abnormalities or delays in physiological development
185	Autism Spectrum Disorder	Diagnosis of autism spectrum disorder
74	Behavioral, sensory, and intellectual impairment	Any diagnosis that related to patient behavior, sensory processing, or intellectual impairments
7	Cardiac impairment	Any diagnosis that indicated a cardiac impairment or cardiac malformation
101	Cerebral palsy	Any diagnosis that included cerebral palsy
144	Congenital abnormalities	Any diagnosis indicating congenital abnormalities including syndromes, chromosomal abnormalities, and malformations
9	Delayed milestones	Any diagnosis indicating delayed developmental milestones including gross motor, fine motor, and language
81	Feeding difficulties	Any diagnosis that indicated feeding difficulties
195	Movement disorder	Any diagnosis of abnormal movement including hypotonia, ataxia, and apraxia
220	Musculoskeletal impairment	Any diagnosis that indicated impairment of the musculoskeletal system
50	Non-congenital insult to the brain	Any diagnosis that indicated a non-congenital insult to the brain, such as stroke or cancer
44	Other	Any diagnosis that did not fit into any of the defined diagnosis groups
34	Prematurity or low birth weight	Any diagnosis that indicated a premature birth or low birth weight

### THERAPIST SURVEYS

During the specified time frame of the study, therapists (n= 9) elected to work from home one day per week, during which they treated exclusively telerehabilitation patients, either via video or phone. These therapists completed a work-from-home survey at the conclusion of each remote workday. Therapists who utilized a hybrid approach while remaining on-site throughout the week were excluded from this survey process.

The survey to gauge the successes of our sessions delivered through telerehabilitation was developed by Developmental Physical and Occupational Therapy Program Managers. It was delivered to physical and occupational therapists via the Research Electronic Data Capture (REDCap) tools hosted at Nationwide Children's Hospital (Harris et al., 2009, 2019). REDCap is a secure, web-based software platform designed to support data capture for research studies, providing (1) an intuitive interface for validated data capture; (2) audit trails for tracking data manipulation and export procedures; (3) automated export procedures for seamless data downloads to common statistical packages; and (4) procedures for data integration and interoperability with external sources (Harris et al., 2019). The survey had a total of 20 questions: nine yes/no, five multiple choice, and six open-ended. The questions addressed three domains of telerehabilitation: (1) technology, (2) clinical care quality, and (3) caregiver involvement.

### DATA ANALYSIS

IBM SPSS version 23.0 was used for statistical analysis. Means, standard deviations, medians, and IQR are used to describe patient age. Percentages are used to describe diagnosis group, language preference, Centers for Disease Control (CDC) county level for all patients, and therapist survey results.

## RESULTS

## RAPID IMPLEMENTATION OF TELEREHABILITATION

Physical and occupational therapists were provided education on telerehabilitation at seven different outpatient rehabilitation settings within the same pediatric hospital system. From April 1, 2020 to April 30, 2020, there were a total of 1,352 unique patient visits (*n*=514 patient visits for physical therapy; *n*=838 patient visits for occupational therapy) (Figure 1). Patients seen in the clinic were *n*= 224 (16.6%) and patients seen via telerehabilitation were *n*= 1,128 (83.4%). There were 938 video visits and 150 telephone encounters.

**Figure 1a:**
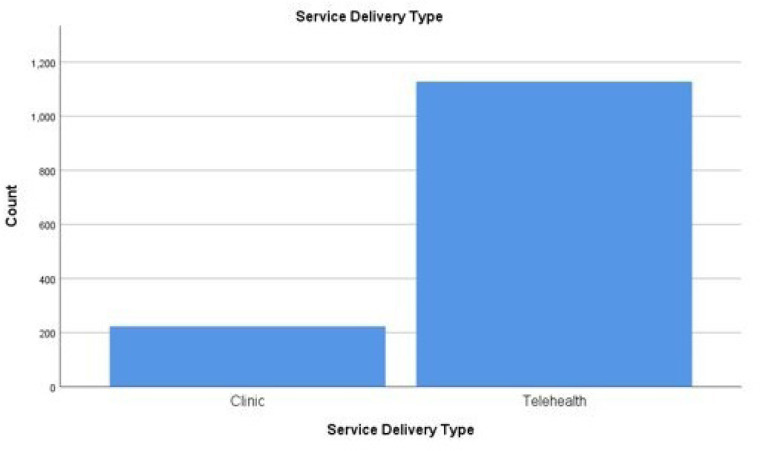
Service Delivery Type

*Figure 1a.* Bar graph of count of patients seen in the clinic (left) or via telerehabilitation (right). n= 224 patients were seen in the clinic and n= 1,128 patients were seen via telerehabilitation.

**Figure 1b:**
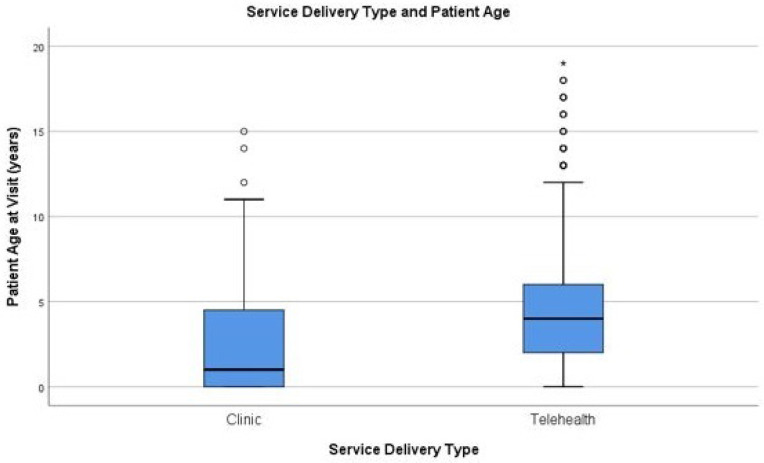
Service Delivery Types and Patients Ages

*Figure 1b.* Boxplot of service delivery type (clinic on the left or telerehabilitation on the right) by age of the patient at the time of the encounter. The median age of patients seen in the clinic was 1.0-year-old with an IQR of 5.0 years (2.5±.22 years), and the median age of patients seen via telerehabilitation was 4.0 years old with an IQR of 4.0 years (4.6±.10 years).

## DEMOGRAPHICS OF PATIENTS RECEIVING REHABILITATION SERVICES DURING COVID-19

The median age of patients receiving physical or occupational therapy was 4.0 years old with an interquartile range (IQR) of 5.0 years (4.3±3.5 years) at the time of visit. The median age of patients seen in the clinic was 1.0-year-old with an IQR of 5.0 years (2.5±.22 years) and the median age of patients seen via telerehabilitation was 4.0 years old with an IQR of 4.0 years (4.6±.10 years) (Figure 1). The median age of patients seen by physical therapy was 2.0 years old with an IQR of 4.0 years (3.0±.16 years), which was a lower median age than occupational therapy. Patients seen by occupational therapy had a median age of 5 years old with an IQR of 4 years (5.0±.12 years).

**Figure 2:**
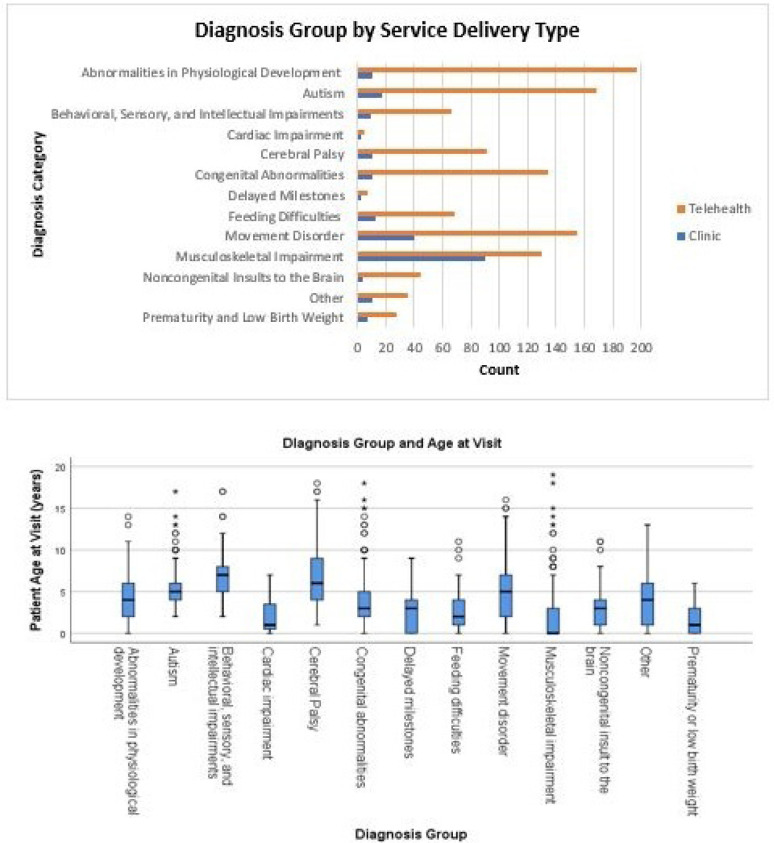
Diagnosis Group by Service Delivery Type and Age

Patients with musculoskeletal impairments had the highest percentage of in-person clinic visits (41.0%), followed by cardiac impairment (28.6%), autism spectrum disorder (22.2%), and delayed milestones (22.2%). Patients with prematurity or low birth weight had the highest percentage of telerehabilitation visits (95.2%), followed by feeding difficulties (93.1%), behavioral, sensory, and intellectual impairment (91.8%), and congenital abnormalities (84.0%) (Figure 2).

*Figure 2a.* Bar graph of count of service delivery type by diagnosis group. The proportion of children in each diagnosis group receiving telerehabilitation (orange) and in-person clinical care (blue). Patients with musculoskeletal impairments had the highest percentage of in-person clinic visits (41.0%). Patients with prematurity or low birth weight had the highest percentage of telerehabilitation visits (95.2%). *Figure 2b.* Boxplot of diagnosis group by age. The patient diagnosis group with the youngest median age was musculoskeletal impairments (0 years old with an IQR of 3 years; 2.06±.23 years). The oldest patient diagnosis group was patients with a behavioral, sensory, and intellectual impairment (7 years old with an IQR of 3 years; 7.05±.18 years old).

The patient diagnosis group with the youngest median age was musculoskeletal impairments (0 years old with an IQR of 3 years; 2.06±.23 years) followed by prematurity or low birth weight (1 year-old with an IQR of 3 years; 1.65±.28 years), cardiac impairment (1-year-old with an IQR of 4 years; 2.29±.55 years). The oldest patient diagnosis group was patients with a behavioral, sensory, and intellectual impairment (7 years old with an IQR of 3 years; 7.05±.18 years old), followed by autism (5 years old with an IQR of 2 years; 5.34±.18 years old), and cerebral palsy (6 years old with an IQR of 5 years; 6.87±.40 years old) (Figure 2).

There were 20 different language preferences reported. Most patients and families in this cohort designated English as their language preference (90.9%), followed by Spanish (3.3%), Nepali (1.3%), Somali (1.3%), Arabic (0.7%), Amharic (0.4%), American Sign Language (0.2%), and other (1.9%). Of the English-speaking patients, 15.8% were seen in the clinic. Patients who spoke Somali had the highest percentage of clinic visits (38.9%), followed by patients who spoke Nepali (29.4%), Arabic (10.0%), Amharic (0%), and American Sign Language (0%) (Figure 3a and Figure 3b).

**Figure 3a:**
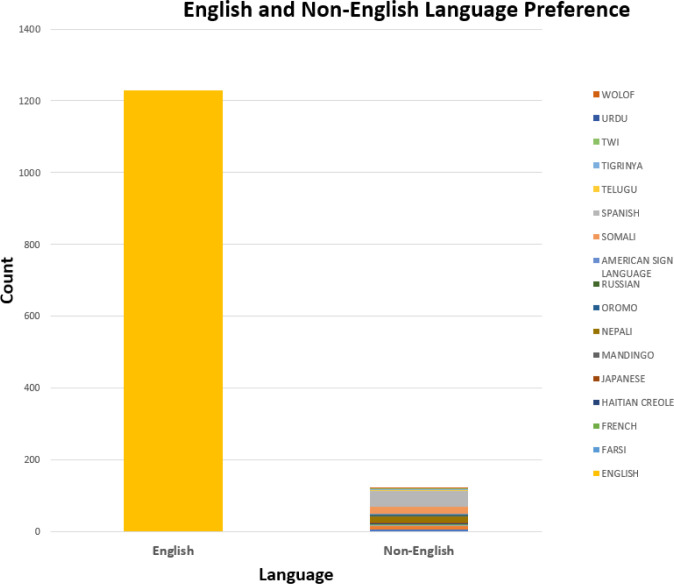
Language Preferences of Patients

*Figure 3a.* English (90.9%) and non-English language (9.1%) preferences for patients receiving services in the clinic and via telerehabilitation.

**Figure 3b:**
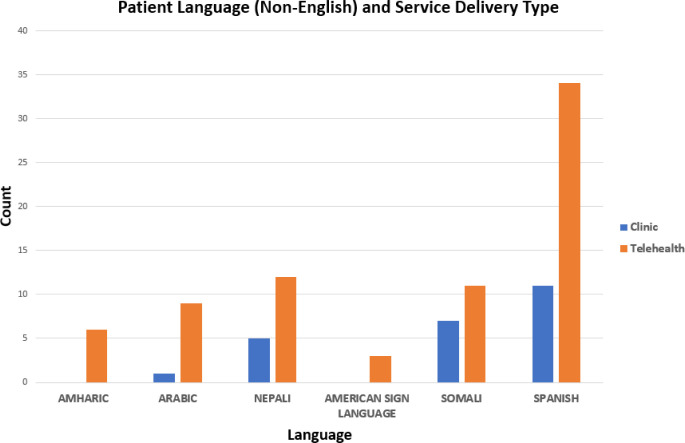
Patient Language (Non-English) and Service Delivery Type

*Figure 3b.* Most non-English speaking patients were seen via telerehabilitation.

Each of the six levels of urban-to-rural classifications was represented in this cohort. Most patients were classified as living in a large central metro (61.0%), followed by large fringe metro (24.9%), micropolitan (6.9%), small metro (6.4%), noncore (0.5%), and medium metro (0.2%).

## THERAPIST SURVEY

The technology questions answered by the therapists are presented in Table 2. The results showed an 89.2-99.7% positive responses regarding technology. Most therapists (69.1%) reported that telerehabilitation sessions were equal in clinical care to in-person clinic visits; 13.4% reported that telerehabilitation was better, and 17.5% reported that telerehabilitation was not equal to in-person clinic visits. In addition, most therapists (92.5%) reported that caregivers were present and actively participating in the telerehabilitation session, 3.6% reported that caregivers were observing but not participating, and 3.9% reported caregivers were present but occupied in the background.

**Table 2:** Technology Questions and Therapist Response

Question	Therapist response “yes” (%)	Therapist response “no” (%)
Were there any hardware challenges for the clinician?	1.9%	98.1%
Were there any connectivity (WIFI) challenges for the clinician?	1.7%	98.3%
Were there any application/EPIC/MyChart challenges for the clinician?	1.9%	98.1%
Were there any hardware challenges for the family?	3.6%	96.4%
Were there any connectivity (WIFI) challenges for the family?	10.8%	89.2%
Were there any application/EPIC/MyChart challenges for the family?	6.1%	93.9%
Did clinician have access to all materials needed for this session?	95.3%	4.7%
Was an interpreter used during this session?	3.6%	96.4%
Was an interpreter needed but not obtained?	0.3%	99.7%

## DISCUSSION

Rapid implementation of telerehabilitation was critical during the COVID-19 pandemic for pediatric developmental therapies to continue to provide safe and high-quality clinical services to vulnerable populations (Ben-Pazi et al., 2020). Our study sought to describe the rapid implementation of telerehabilitation, describe demographic characteristics of patients who continued in-person services and those who received telerehabilitation, and evaluate therapists' perceptions of telerehabilitation for physical and occupational therapy at a large, pediatric hospital system in the Midwest, United States.

Telerehabilitation was successfully and rapidly implemented at Nationwide Children's Hospital for developmental occupational and physical therapies. Implementation of telerehabilitation was completed through a multidisciplinary team approach that addressed (1) technology, billing, and documentation, (2) messaging to staff, caregivers, and families, (3) formal and informal educational opportunities, and (5) refinement of services provided through telerehabilitation through feedback from staff and families. Utilizing both telerehabilitation and in-person models of care, services were maintained for a diverse group of patients during the COVID-19 pandemic. Patients seen during this period were (1) 0-18 years old, (2) had 240 unique referral diagnoses, (3) had 20 different language preferences, and (4) represented all county-level populations as categorized by the CDC. These results highlight the usefulness of offering telerehabilitation to maximize patient encounters and continue vital services during a public health crisis, as well as presenting opportunities for future utilization.

Most patients were seen through telerehabilitation (83.4%) at our institution in the early months of the COVID-19 pandemic. Some patients did continue with in-person clinical care; specifically, younger patients and patients with musculoskeletal conditions were more often seen in the clinic. This likely occurred due to our hospital therapy recommendations of who should continue in-person clinical care. These recommendations were developed based on clinical expertise, suggesting that patients under 1-year-old, with torticollis, undergoing serial casting, without access to technology, or who were post-surgery should continue in-person clinical care. We propose, based on clinical expertise that these patients may benefit from continued in-person clinical care because hands-on therapeutic techniques are deemed critical for successful treatment.

Although our recommendations for in-person clinical care were for all patients less than 1-year-old, patients with prematurity or low birth weight (median age of 1-year) had the highest percentage of telerehabilitation visits (95.2%). This population is especially vulnerable in the first year of life, and caregivers/families may have opted for telerehabilitation to promote safety and social distancing. This finding emphasizes the importance of allowing the decision for the model of care to be determined by the caregivers and families of the patients. Parents and families should be educated regarding benefits of and barriers to telerehabilitation to assist in making the decision for the best model of care for their child.

Of the patients seen in-person, more spoke Somali or Nepali than English. No other confounding factors were controlled for, so we cannot say whether these patients were seen in the clinic due to their language preference. However, it is important to consider language when implementing services through telerehabilitation and educate patients, families, and therapists on how to request or obtain an interpreter if needed. In our study, 99.7% of therapists reported that an interpreter was present during the session when needed, but future work should be done to determine whether language is a barrier to efficacious telerehabilitation.

Therapists also reported minimal technology barriers and a high percentage of clinical care quality and caregiver/parent involvement during telerehabilitation treatment sessions. Therapists reported telerehabilitation care to be equal or better than in-person clinical care at 82.5%. In addition, 92.5% of therapists stated that the caregiver/parent was actively involved and participating during the telerehabilitation session. These results highlight the role that telerehabilitation may play in providing therapists more opportunities to understand the patient's home environment and transfer skills from the therapy gym to everyday context. It also may provide ample opportunity for parent/caregiver coaching to increase therapeutic opportunities at home.

## LIMITATIONS AND FUTURE DIRECTIONS

There are several limitations that should be noted for this study. First, the data from this study only included patients who were being seen for developmental occupational and physical therapies at a large, multisite pediatric hospital system in the Midwest, United States in April 2020. This is important to state as it limits generalizability of the results to other disciplines or hospital systems. Second, due to the study design (retrospective, observational), we cannot comment on the effectiveness of therapies provided through telerehabilitation or statistically compare the differences between patients seen via telerehabilitation and in-person clinical care. Future research should focus on understanding the effectiveness of telerehabilitation compared to in-person clinical care and determine which populations would best be served by each model of care.

## CONCLUSION

Telerehabilitation will likely not replace traditional in-person clinical care for all patient populations but should be considered as an important adjunct or additional model of care post-pandemic (American Physical Therapy Association, 2019). The results from this study suggest that implementation of telerehabilitation is feasible, can be offered to a wide diversity of patients and families, and is generally accepted by occupational and physical therapists as an effective model of care for pediatric patients.
