# Errata

**DOI:** 10.5195/ijt.2021.6386

**Published:** 2021-06-22

**Authors:** Ellen R. Cohn

Correction to the Metadata for: Bican, R., Christensen, C., Fallieras, K., Sagester, G., O’Rourke, S., Byars, M., & Tanner, K. (2021). Rapid Implementation of Telerehabilitation for Pediatric Patients During COVID-19. *International Journal of Telerehabilitation, 13*(1). https://doi.org/10.5195/ijt.2021.6371

The affiliation for each author was incorrectly stated as: Physical Medicine & Rehabilitation, Children's Minnesota, Minneapolis, Minnesota, USA

The correct affiliation for each author is: Clinical Therapies, Nationwide Children's Hospital, Columbus, Ohio, USA

The metadata for the original article has been corrected.

